# Identification of Shield Tunnel Segment Joint Opening Based on Annular Seam Pressure Monitoring

**DOI:** 10.3390/s24123924

**Published:** 2024-06-17

**Authors:** Hongbin Xu, Qucheng Liu, Bingtian Li, Chuanrui Guo

**Affiliations:** 1 College of Civil and Transportation Engineering, Institute of Urban Smart Transportation & Safety Maintenance, Shenzhen University, Shenzhen 518060, China; xuhongbin@semi.ac.cn (H.X.); 2110474114@email.szu.edu.cn (Q.L.); 2 National Key Laboratory of Green and Long-Life Road Engineering in Extreme Environment (Shenzhen), Shenzhen University, Shenzhen 518060, China; 3 China Academy of Railway Sciences Co., Ltd., Beijing 100081, China

**Keywords:** shield tunnel, joint opening identification, piezoelectric film, contact pressure

## Abstract

Tunnels for subways and railways are a vital part of urban transportation systems, where shield tunneling using assembled segmental linings is the predominant construction approach. With increasing operation time and varying geological conditions, shield tunnels usually develop defects that compromise both structural integrity and operational safety. One common issue is the separation of segment joints that may cause water/mud penetration and corrosion. Existing inspection strategies can only detect openings after their occurrence, which cannot provide early warnings for predictive maintenance. To address this issue, this work proposes a multi-point seam contact pressure monitoring method for joint opening identification. It first derived the theoretical correlation between contact pressure distribution and segment opening; then, a finite element model was established to explore the stress and deformation responses under combined axial and bending loads. Finally, multi-point piezoelectric film sensors were implemented on a scaled segment model to validate the theoretical and numerical analyses. Results indicate that the multi-point monitoring method can effectively identify opening amounts at the segment joints with an average error of 8.8%, confirming the method’s feasibility. These findings support the use of this monitoring technique for early detection and assessment of joint openings in shield tunnels.

## 1. Introduction

In the past decades, many countries have invested in urban rail transit systems, including subways, as a primary mode of urban transportation. Shield tunnel construction has become extensively used in subway projects due to its minimal weather dependency, low environmental impact, and high efficiency [1]. However, as service time increases, the prefabricated segments used in shield tunnels are prone to issues such as joint openings and misalignments, water leakage, and other types of deformation, which threaten structural safety and can lead to accidents [2,3,4]. Therefore, structural health monitoring and maintenance for shield tunnels is crucial.

During the operation period, various factors can undermine shield tunnel conditions, such as the quality of the segments, changes in geological conditions, maintenance oversights, and the impacts of external construction. As an assembled structure, the long-term load-bearing parts of a shield tunnel are primarily the segment joints that are connected by bolts. Since most defects initially appear at these joints, the condition of the segment joints significantly affects the tunnel’s structural integrity and safety. Common joint defects include joint openings, misalignments, water seepage, etc. Among these, joint openings are the most dangerous since they can cause water penetration and strength reduction. Even though inspection techniques such as 3D laser scanning and machine vision have been widely used in shield tunnels [5], they can only reveal the defects after their occurrence, which means the defect has already existed for a long time and the ideal maintenance timing might have been missed. Therefore, the development of a monitoring technology that can provide early warnings for joint openings before or shortly after openings occur—thus reducing maintenance costs—is urgent.

In the past years, researchers have proposed a real-time convergence monitoring system for straight shield tunnels using wireless inclinometers, optimizing sensor placement for accuracy, and validating their applicability with field cases [6,7,8,9]. Tan et al. have used hydrometers and temperature sensors to measure pressure and temperature changes at underwater tunnel segment joints, indirectly assessing joint opening changes through model predictions [10]. Liu et al. have explored the performance of ultra-high-performance concrete (UHPC) reinforced shield tunnel linings through full-scale load-bearing tests, measuring joint opening amounts with electronic displacement meters [11]. Zhang et al. have suggested using MEMS inclinometers to measure segment rotation angles to calculate the maximum width of joint openings, finding measurement accuracy superior to that of displacement meters around the joint area, providing insights into automating monitoring systems [12]. However, the above methods mainly detect deformations or openings after they have occurred.

The process of annular seam opening and the associated changes in joint contact pressure are directly correlated [13,14,15] and are theoretically validated through longitudinal beam-spring calculation models in the literature [16,17,18]. Therefore, by multi-point monitoring of annular seam contact pressure, the status of the seam can be obtained and thus the joint opening amounts can be identified, reducing error and uncertainty [19,20]. Such monitoring and identification techniques have not been investigated before.

To address the above issue, this work proposes a multi-point contact pressure monitoring method at the annular seam for joint opening identification. The method first establishes the correlation between annular seam contact pressure and joint opening amounts through theoretical analysis and numerical simulation, which can be utilized to identify the joint opening through contact pressure monitoring data. Experimental tests and results indicate that the proposed method can identify the joint opening amount effectively and can thus provide an early warning for joint openings for the safe operation of shield tunnels.

## 2. Theoretical Analysis of the Correlation between Joint Opening and Seam Contact Pressure

Based on the opening conditions of the joint during the longitudinal bending deformation of a segment, the force and deformation states at the seams can be categorized into three types: fully closed, partially open, and fully separated [21,22,23]. This work primarily investigates the partially open condition. 

**Figure 1:**
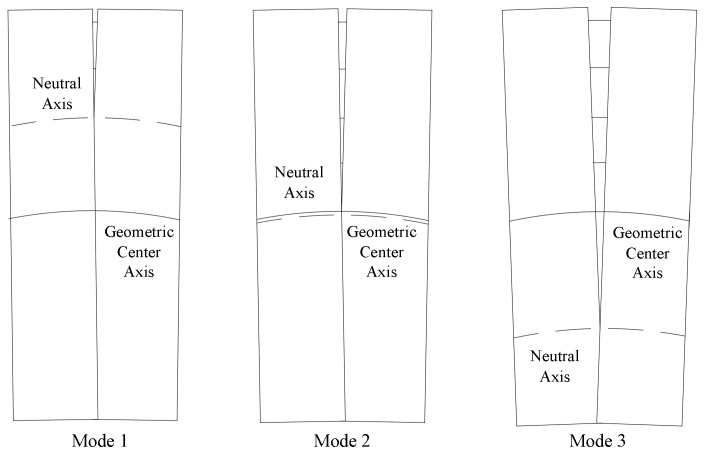
Three deformation modes during the opening process.

Throughout the opening process, the neutral axis moves across the full height of the segment as the opening amount increases from the open side towards the geometric center axis until reaching the edge of the seam on the opposite side. As shown in Figure 1, the deformation modes above and below the geometric center axis are referred to as Modes 1 and 3, respectively, while alignment with the geometric center axis is referred to as Mode 2. Based on the longitudinal axial force characteristics and ignoring shear effects, the force states at the seams can be divided into three types: pure bending, compressive bending, and tensile bending. These force states are not present in all deformation modes; Mode 1 does not exhibit pure or tensile bending, Mode 2 represents the limit deformation mode for pure and tensile bending but is not actually achievable, and Mode 3 could exhibit all three force states.

As compressive bending is the most likely force state among the three deformation modes, calculations are initially focused on this case. Axial force is considered as pressure, with bending moments causing tension on the upper part and compression on the lower part of the segment. The deformation involves opening at the upper part of the annular seam. Due to the tight connection between segment rings in the compressed region, the contact pressure at the seam is approximately equal to the internal pressure of the concrete.

**Deformation Mode 1:** With a small amount of joint opening and the neutral axis located above the geometric center axis, most sections are under compression. The upper part of the segment ring opens under the influence of bending moments, with bolts and segments simultaneously bearing tension while the lower part of the segment bears compression; bolts do not participate in the force.

**Figure 2:**
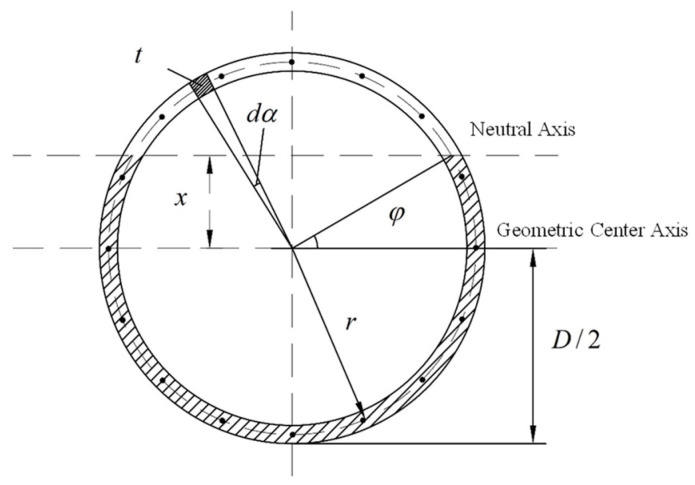
Force distribution on seam under mode 1.

**Figure 3:**
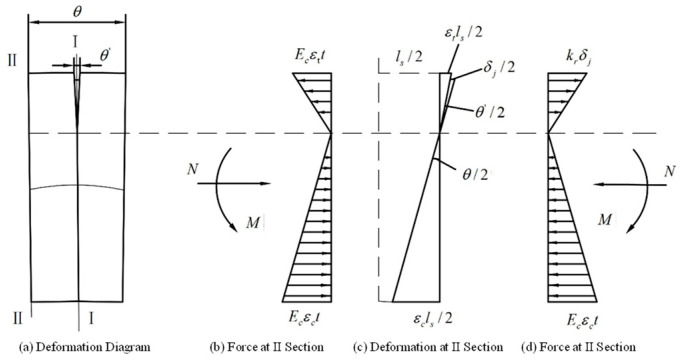
Deformation and force analysis under mode 1.

At this point, the position of the neutral axis and the section force situation are shown in Figure 2. The angle φ is illustrated, defined as the angle between the line from the center of the segment ring to the neutral axis and the horizontal axis at the intersection with the ring wall center. Considering the symmetry between the two segment rings, half of the width of the two segment rings and the connecting bolts are analyzed. The bending deformation in this region is shown in Figure 3a, where the geometric dimensions of this area are symmetric with respect to external forces about section I. The left side of this region is analyzed, and deformation coordination equations and mechanical equilibrium equations are established and represented by Equations (1)–(4). The force and deformation on cross-sections I and II of the structure are illustrated in Figure 3b–d.

Deformation compatibility equation:

(1) $$\varepsilon_{c}\cdot \frac{l_{s}}{2}=\left(R+x\right)\cdot \frac{\theta }{2}$$

Equation of equilibrium:

(2) $$\frac{r-x}{R-x}\cdot \varepsilon_{t}\cdot \frac{l_{s}}{2}+\frac{\delta_{j}}{2}=\left(r-x\right)\cdot \frac{\theta }{2}$$

(3) $$2\frac{E_{c}\varepsilon_{c}t}{R+x}\int_{-\frac{\pi }{2}}^{\varphi }\left(x-r\sin\alpha \right)rd\alpha -N=\;2\frac{E_{c}\varepsilon_{t}t}{R-x}\int_{\varphi }^{\frac{\pi }{2}}\left(r\sin\alpha -x\right)rd\alpha =2\frac{k_{r}\delta_{j}}{r-x}\int_{\varphi }^{\frac{\pi }{2}}\left(r\sin\alpha -x\right)rd\alpha$$

(4) $$2\frac{E_{c}\varepsilon_{c}t}{R+x}\int_{-\frac{\pi }{2}}^{\varphi }{\left(x-r\sin\alpha \right)}^{2}rd\alpha +2\frac{E_{c}\varepsilon_{t}t}{R-x}\int_{\varphi }^{\frac{\pi }{2}}{\left(r\sin\alpha -x\right)}^{2}rd\alpha =M+N\cdot x$$

*x* represents the distance from the neutral axis on the annular seam contact surface to the horizontal geometric center axis, with *x* = *r*sin*ϕ*; *ϕ* is the angle of the neutral axis position, where *ϕ* ϵ [−*π*/2,*π*/2]; *R* is half of the outer diameter of the segment ring, *R* = *D*/2; $\varepsilon_{t}$ and $\varepsilon_{c}$ are the longitudinal tensile and compressive strains at the upper and lower parts of the segment ring on the contact surface, respectively; *δ*_*j*_ is the elongation of the upper bolts on the contact surface; *r* is the distance from the bolt position to the center of the cross-section; *k*_*r*_ is the tensile stiffness per line of the bolts at the joint between rings, calculated as *k*_*r*_ = *nk*_*j*_/(2*πr*), where *k*_*j*_ is the tensile stiffness of a single bolt; *E*_*c*_ is the modulus of elasticity of the concrete in the segment.

By combining Equations (1)–(4), Equation (7)—involving longitudinal axial force *N*, bending moment *M*, and the angular rotation *θ* of the two segment rings—is derived, along with Equation (8) for the position of the neutral axis. Here, a four-variable expression (5) that includes angle *ϕ* of the neutral axis is introduced to simplify the expression of results.

(5) $$\left\{\begin{array}{l} A=\cos\varphi +\sin\varphi \left(\varphi -\frac{\pi }{2}\right)\\ B=\cos\varphi +\sin\varphi \left(\varphi +\frac{\pi }{2}\right)\\ C=\left(\frac{\pi }{2}-\varphi \right)\left(\frac{1}{2}+\sin^{2}\varphi \right)-\frac{3}{4}\sin2\varphi \\ D=\left(\frac{\pi }{2}+\varphi \right)\left(\frac{1}{2}+\sin^{2}\varphi \right)+\frac{3}{4}\sin2\varphi \end{array}\right.$$

Combining the deformation correlation below:

(6) $$\frac{\theta }{2}=\frac{\theta^{\prime }}{2}+\frac{\varepsilon_{t}l_{s}}{2\left(R-x\right)}$$

The expressions for the angular rotation *θ*′ between the annular seams and the angular rotation *θ* of the two segment rings can be derived:

(7) $$\left\{\begin{array}{l} \theta =\frac{Nl_{s}\left(E_{c}t+k_{r}l_{s}\right)}{2{r^{2}E}_{c}t\left[\left(k_{r}l_{s}+E_{c}t\right)B-k_{r}l_{s}A\right]}\\ \theta^{\prime }=\frac{E_{c}t\cdot \theta }{k_{r}l_{s}+E_{c}t} \end{array}\right.$$

Position of the neutral axis:

(8) $$\frac{Nr\cdot k_{r}l_{s}\left(AD+BC\right)}{\left(k_{r}l_{s}+E_{c}t\right)B-k_{r}l_{s}A}=\left(M+Nr\sin\varphi \right)B-Nr\cdot D$$

Due to varying degrees of opening and pressure at different positions on the annular seam contact surface, the upper segments on the open side are separated from each other, resulting in zero contact pressure at the seam. The opening amount decreases as the distance to the neutral axis decreases. On the lower, closed side, the annular seam contact surface is in a compressed state, where the contact pressure increases as the distance to the neutral axis increases, and the opening amount is zero. Given that the contact pressure of the annular seam is distributed vertically in a triangular pattern, a pressure equation for the contact surface at different positions can be obtained:

(9) $$F=E_{c}\varepsilon_{c}t\cdot \frac{x-R\sin\alpha }{R+x}$$

Then, the seam contact pressure expression can be derived:

(10) $$F=\frac{N\left(E_{c}t+k_{r}l_{s}\right)}{2r^{2}\left[\left(k_{r}l_{s}+E_{c}t\right)B-k_{r}l_{s}A\right]}\cdot \frac{x-R\sin\alpha }{R+x}$$

In this work, maximal seam contact pressure and opening are selected for analysis considering simplicity and engineering applications; the opening amount δ at the top of the seam and the bottom contact pressure $F_{max}$:

(11) $$\delta =\varepsilon_{t}l_{s}+\delta_{j}$$

(12) $$F_{max}=E_{c}\varepsilon_{c}t$$

Then the correlation between δ and $F_{max}$ can be expressed as:

(13) $$\delta =\frac{F_{max}l_{s}}{R+r\sin\varphi }\cdot \frac{\left(R-r\sin\varphi \right)k_{r}l_{s}+(r-r\sin\varphi )E_{c}t}{E_{c}t(E_{c}t+k_{r}l_{s})}$$

**Deformation Mode 2:** When the neutral axis aligns with the geometric center axis of the cross-section, the angle of the neutral axis, *ϕ*, equals 0. Substituting this into Equation (7), the expressions for the angular rotation *θ*′ between the annular seams and the angular rotation *θ* of the segment rings are as follows:

(14) $$\left\{\begin{array}{l} \theta =\frac{Nl_{s}\left(E_{c}t+k_{r}l_{s}\right)}{2{\left(rE_{c}t\right)}^{2}}\\ \theta^{\prime }=\frac{E_{c}t\cdot \theta }{k_{r}l_{s}+E_{c}t} \end{array}\right.$$

Correlation between δ and $F_{max}$ can be expressed as:

(15) $$\delta =\frac{F_{max}l_{s}}{R}\cdot \frac{k_{r}l_{s}R+E_{c}tr}{E_{c}t(E_{c}t+k_{r}l_{s})}$$

**Deformation Mode 3:** In this mode, the neutral axis of the annular seam contact surface moves below the geometric center axis. The forces and deformation are similar to those depicted in Figure 3. By changing the sign of *N* in Equations (7) and (8), the function for the angular rotation between annular seams and the equation for the position of the neutral axis in this deformation mode can be derived. Consequently, the relationship between the maximum annular seam contact pressure and the opening amount can be established:

(16) $$\delta =\frac{F_{max}l_{s}}{R+r\sin\varphi }\cdot \frac{\left(R-r\sin\varphi \right)k_{r}l_{s}+(r-r\sin\varphi )E_{c}t}{E_{c}t(E_{c}t+k_{r}l_{s})}$$

So far, the correlation between joint opening amount and seam contact pressure under three different modes have been theoretically established. Parametric study will be conducted using the derived equation together with the numerical simulations in the next section.

## 3. Numerical Simulation

To verify the accuracy of the theoretical analysis, this section uses ABAQUS 2022 finite element software to simulate the stress conditions at the segment ring joints of shield tunnels when bending deformation occurs. It analyzes the distribution of annular seam contact pressure and changes in the opening amount under different combinations of longitudinal axial force and bending moment. The finite element results are extracted and compared with the theoretical analysis to study the similarity in their curve trends and to analyze potential errors and their causes.

### 3.1. Engineering Case of a Cross-River Shield Tunnel for Modelling

**Figure 4:**
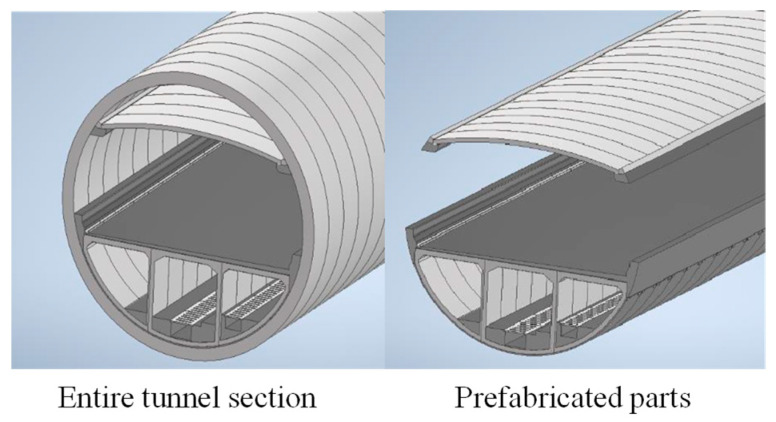
Schematic illustration of the tunnel section.

One shield tunnel under construction in China is chosen for numerical simulation and theoretical analysis. The tunnel is an underwater shield tunnel with a total length of 11.185 km. The designed section and prefabricated segments are shown below (Figure 4):

### 3.2. Finite Element Model of Two Segments and Joints

**Figure 5:**
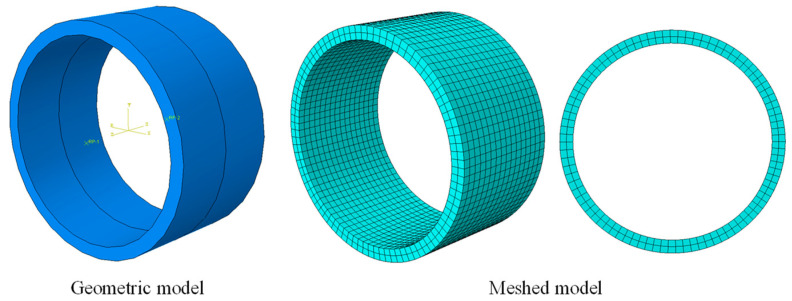
Geometric and meshed model for numerical simulation.

The geometric model and meshed model are shown in Figure 5. Since the main focus is on the distribution of contact pressure in the annular seams during tunnel bending deformation and its relationship with the opening amount, and, given that the overall length of the shield tunnel is much greater than its diameter—approximating a rod-like structure—its longitudinal stiffness is significantly smaller compared to its lateral stiffness. This becomes one of the main factors affecting tunnel structural deformation. Therefore, when constructing the segment model, a complete ring is built by default, binding all segments within the ring to each other, preventing relative displacements and deformations. The specific model parameters are shown in Table 1. 

A surface-to-surface contact relationship is established at the seam contact surface, with contact properties set to normal hard contact allowing for separation after contact, and tangential contact established using a penalty function for friction relationships, adopting a friction coefficient of 0.62 as suggested in literature [24].

### 3.3. Boundary Condition and Loading

**Figure 6:**
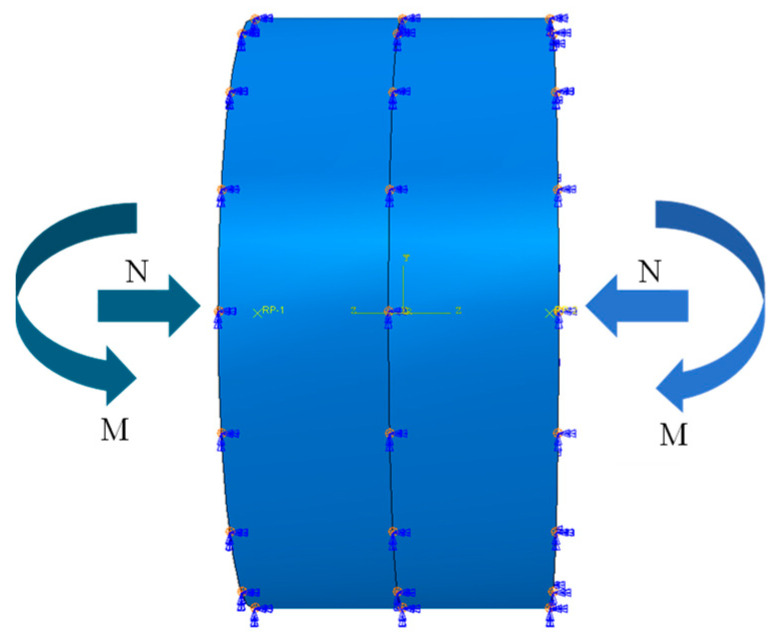
Boundary condition and loads for numerical simulation.

This work investigates the relationship between contact pressure and opening amount at the annular seams of shield tunnels based on a beam-spring model. The method of applying longitudinal axial forces and bending moments is illustrated in Figure 6, achieved by establishing reference points that are coupled with the outer cross-sections of the segment rings on both sides of the tunnel [25]. Concentrated forces and bending moments are then applied at these reference points. Since the applied loads only cause bending within the yz-plane, it is necessary to impose constraints on the exterior of the overall model to prevent rotation within other planes, as well as to apply displacement constraints in the x-direction to prevent lateral movement of the segment rings [26,27,28]. The axial force from 0 to 6000 kN (2000 kN step) and a bending moment from 0 to 30,000 kNm (5000 kNm step) are applied on the model for analysis.

### 3.4. Simulation Results and Comparison with Theoretical Analysis

**Figure 7:**
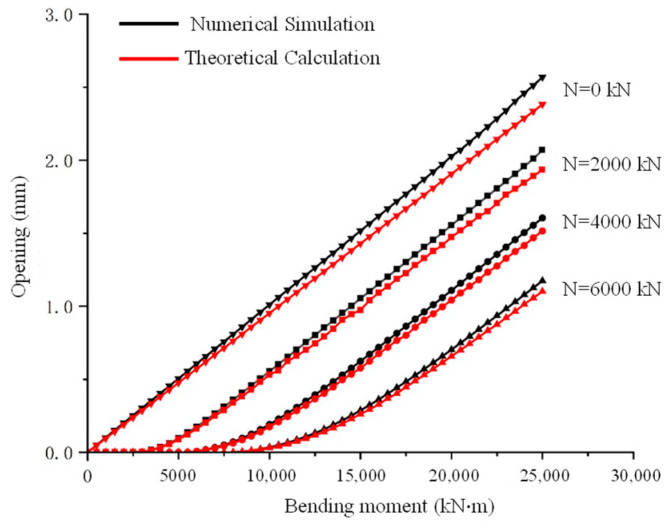
Numerical and theoretical results—comparison of correlation between opening amount and bending moment under various axial forces.

Figure 7 presents a comparison between the finite element and theoretical analysis results of the relationship curves between the annular seam opening amount and bending moment, clearly showing that both methods yield curves with the same trend. As illustrated in Figure 3, Figure 4, Figure 5 and Figure 6, under pure bending conditions (i.e., N = 0), the δ–M curve is a straight line passing through the origin, showing a linear positive correlation, consistent with the conclusions derived in Section 2. Under compressive bending conditions, the δ–M curve corresponding to different axial forces lies below the pure bending curve. Initially, it is a horizontal line passing through the origin, indicating that the contact surface of the seam remains closed until the bending moment increases to the critical moment required for segment opening. The longer the line, the higher the critical bending moment needed to initiate opening, and the later the opening of the segment. The curve graphically shows that the greater the axial compression, the higher the critical bending moment. Once the segments begin to open, the curve gradually becomes a slanted straight line parallel to the pure bending curve.

**Figure 8:**
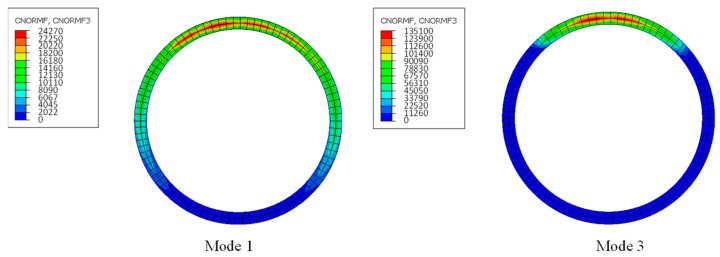
Contact pressure distribution from numerical simulation.

Figure 8 shows the distribution of contact pressure along the annular seams under different deformation modes, showing a symmetrical distribution of contact pressure across the entire contact surface along the vertical geometric center axis. In the open areas, the segment rings are separated from each other, resulting in zero contact pressure at the seams. In the closed areas, the pressure decreases progressively from top to bottom, with the maximum values primarily concentrated around the circular region where the bolts are located. The closer to the neutral axis, the more pronounced the decrease in pressure, until it abruptly drops to zero upon entering the open area.

**Figure 9:**
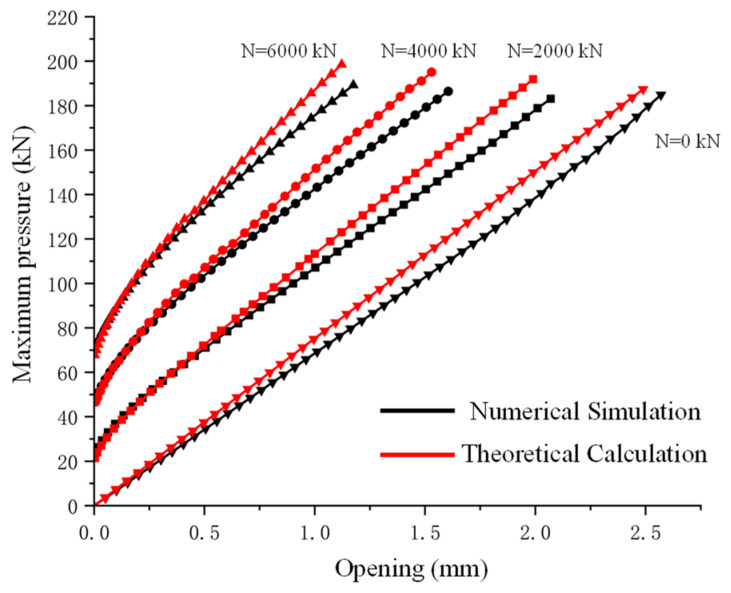
Numerical and theoretical results—comparison of correlation between opening amount and maximum pressure under various axial forces.

Figure 9 shows the relationship curves between the maximum annular seam contact pressure and the opening amount under different load combinations. The graph demonstrates a high consistency between the finite element and theoretical analysis results.

## 4. Experimental Validation

As indicated by the simulation results discussed previously, there exists a nonlinear proportional relationship between the contact pressure of the annular seams and the opening amount in shield tunnel segment rings. However, both the theoretical analysis and numerical simulations are based on ideal calculations under various assumed constraints. To enhance the accuracy of the conclusions and achieve opening amount identification, scaled-down models of the tunnel are fabricated as experimental tests. Through load testing that simulates the bending deformation of shield tunnels, the measured results are used to validate the proposed method for joint opening identification.

### 4.1. Experimental Setup

#### 4.1.1. Scaled Segment Model

**Figure 10:**
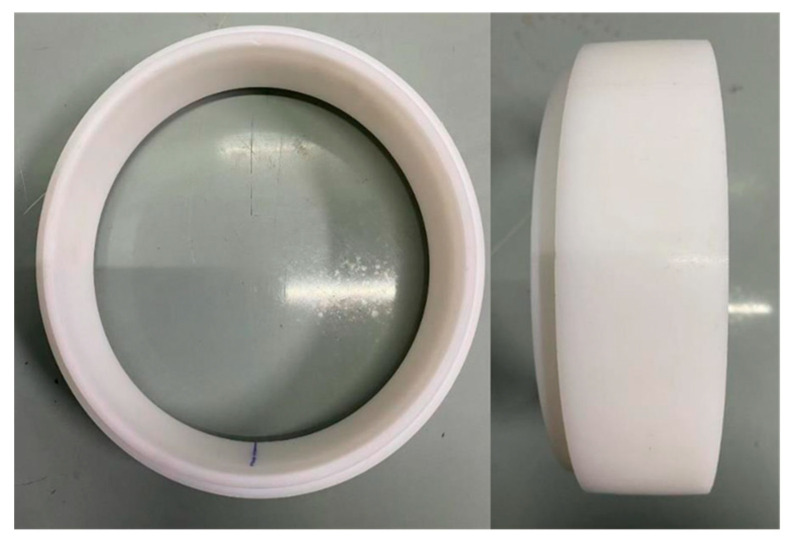
Scaled segment model fabricated by LDPE.

The prototype segment used in real engineering has an outer diameter of 6m, a thickness of 0.35 m, and a ring width of 1.5 m. Based on similarity theory, a scaled model that meets mechanical performance requirements was fabricated [29]. The geometric similarity ratio is 1:30, resulting in a model segment with an outer diameter of 200 mm, a thickness of 11.7 mm, and a ring width of 50 mm. The segment material used is Low-Density Polyethylene (LDPE). LDPE has a density of 0.91–0.93 g/cm^3^ and an elastic modulus of 172 MPa. The inter-joint part of the segments features a mortise and tenon structure, allowing for nested connections, and the segment model is shown in Figure 10.

#### 4.1.2. Monitoring Sensor and Data Acquisition

**Figure 11:**
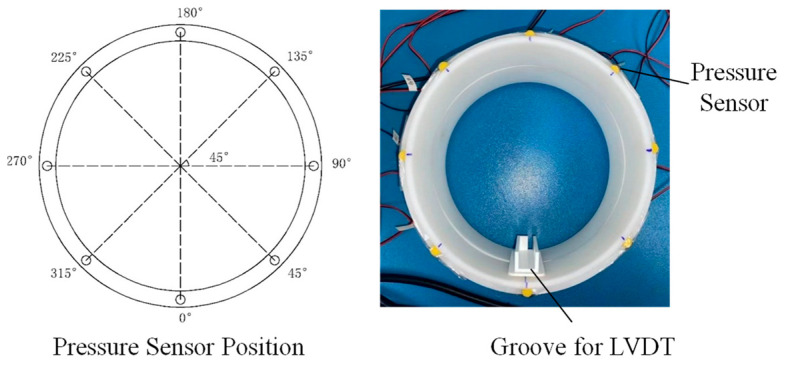
Schematic illustration of sensor deployment.

(1) Seam contact pressure sensor: Since the opening position is unpredictable in the 360-degree range, piezoelectric thin film pressure sensors are arranged at 45° intervals along the cross-section at the connection points of the model segment rings (as shown in Table 2). These sensors measure the contact pressure values and their changes at various measurement points when the tunnel undergoes different degrees of opening [30]. The sensor output signal wires are connected to a data acquisition device and accompanying equipment. The Dewesoft data acquisition device (Sirius-HD) is used to record and analyze data in real-time. The arrangement of the sensors is depicted in Figure 11.
(2) Joint opening sensor: The middle part of the model tunnel, consisting of two segment rings, is selected as the subject of the experiment. Due to the bending deformation adopted in the experiment, which causes compression at the upper part of the segment rings and opening at the lower part, a stop block and a linear displacement sensor (referred to as LVDT, see Table 1) are fixed inside at the bottom of each segment. This setup ensures that when the segment rings are connected and closed, the stop block can push the rod back into the sensor to reset it, thereby measuring the opening amount of the annular seam. The opening condition is verified and data are recorded using an external digital display.
(3) Axial force sensor: The magnitude of the longitudinal axial force applied to the segment model by the control baffle is monitored using ring-shaped force sensors placed at the bolt washers. The sensors are connected to a data acquisition device via signal wires to collect and record data in real time. The sensor setup is shown in Table 1 and Figure 11.

### 4.2. Loading Setup

**Figure 12:**
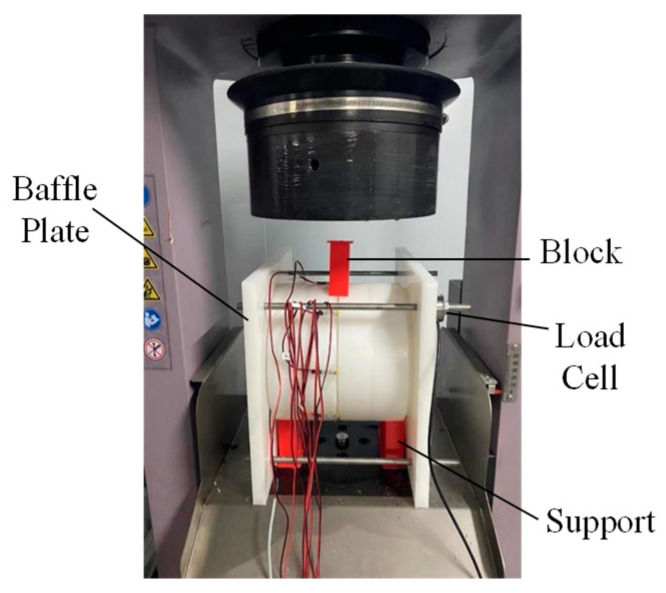
Loading setup.

To simulate the bending deformation of a shield tunnel that causes the segments to open, this experiment employs a loading strategy similar to three-point bending. Two baffle plates are added to the sides of the model tunnel, connected and secured with four bolts using nuts to control the magnitude of the longitudinal axial force. Supports are placed under the segment rings near the baffles to ensure that the tunnel does not slide down the outer walls of the baffles during compression. Above the connection point of the middle segment rings of the model tunnel, blocks are placed to assist the press in applying a concentrated load. Both the supports and blocks are manufactured using 3D printing. The actual test setup is shown in Figure 12.

### 4.3. Test Results

**Figure 13:**
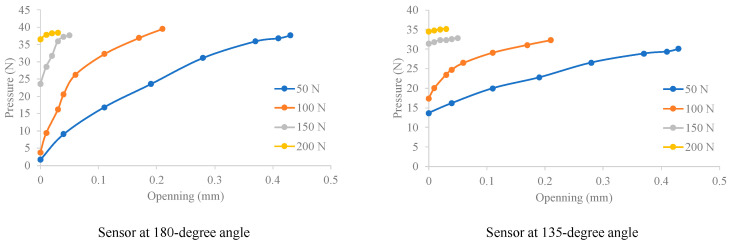
Measured pressure at 180 and 135-degree sensors versus joint opening under different axial forces.

The experimental conditions involved applying axial forces of 50 N, 100 N, 150 N, and 200 N on both sides of the model, with a concentrated central load *P* increasing in increments of 200 N from 0 to 2000 N, directed downwards to induce opening at the lower part of the segments. The pressure changes at 180 and 135-degree measurement points on the contact surface between the two middle ring segments were monitored and recorded. The relationship curves between the annular seam opening amount and the pressure changes are shown in Figure 13.

It can be observed that the maximum contact pressure at the top of the segment ring contact surface (at 180°) increases with the opening amount. The curves corresponding to larger axial forces require a higher critical bending moment for opening, hence a later opening time. Therefore, the vertical lines corresponding to the pressure at the initial stage before the segment opens are longer under higher axial forces. As depicted in the graph, the curve for an axial pressure of 150 N starts higher than those for 50 N and 100 N, with an initially higher maximum annular seam contact pressure at opening. The *F*−*δ* curves at 135° are also monotonically increasing convex curves, with a slightly slower growth rate than at 180°.

## 5. Joint Opening Identification Results and Discussion

**Figure 14:**
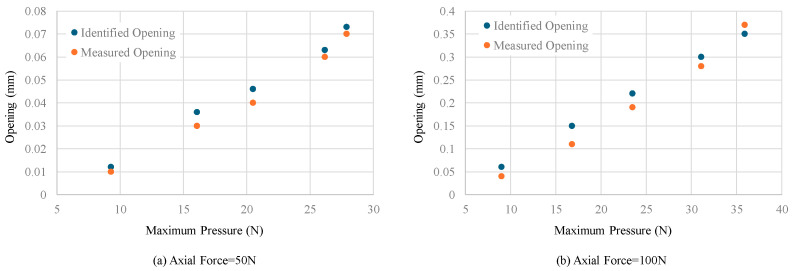
Joint opening identification results using maximum pressure and comparison with LVDT measured results.

To identify the joint opening, the maximum contact pressure was first used in Equations (13), (15), and (16). The position of neutral axis was obtained from the multi-point pressure data. The identified opening results were compared with the LVDT measured results in Figure 14. The comparison indicates that the maximum contact pressure can identify the joint opening well under different external loads. The average identification error under 50 N axial force is 19% and under 100 N is 12%.

Considering that starting solely from the maximum annular seam contact pressure results in low data utilization and potentially compromised accuracy when determining the change in the annular seam opening, and that once the sensor at the maximum pressure point is damaged, data cannot be collected, making it difficult to proceed with opening identification. Additionally, beneath the geometric center axis, the opening speed is faster, and the measured pressure increases slightly before quickly returning to zero. Therefore, more data points can be utilized to calculate the equivalent pressure, *F*_*eq*_, located at the midpoint between the maximum annular seam contact pressure and the average contact pressure, using the contact pressures at 135°, 180°, and 225° on the joint annular seam. The pressures at these three points primarily show an increasing trend during the segment opening process, avoiding premature zeroing of pressure, which yields better results. Based on the moment equilibrium of all forces $M_{eq}=M_{max}+\bar{M}$, *F_eq_* can be expressed as:

(17) $$F_{eq}=\frac{2(\bar{F}\mathrm{Rsin}\theta +F_{max}R)}{\mathrm{Rsin}\theta +R}$$

**Figure 15:**
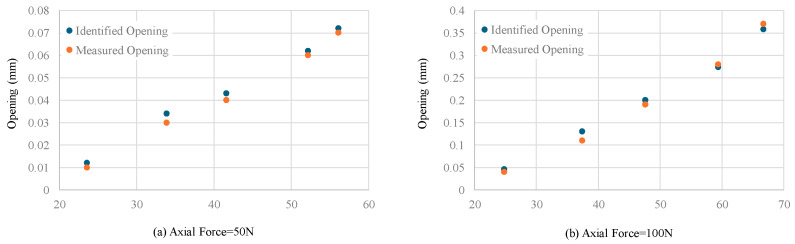
Joint opening identification results using multi-point pressure data and comparison with LVDT measured results.

The joint opening amount can then be identified through the equivalent pressure. The identification results and comparison with the LVDT measured results are shown in Figure 15. The average identification errors are improved to 9.4% for 50 N and 8.8% for 100 N axial force.

## 6. Conclusions

This work proposes a multi-point contact pressure monitoring method for annular seams to identify joint openings, which focuses on the relationship between the contact pressure of segment ring seams and the opening amount in shield tunnels. Theoretical analysis, numerical simulation, and experimental verification were conducted to validate the proposed method. The correlation between contact pressure and opening amount under different loads was derived, finite element models were established for simulation, and the results were compared with theoretical analyses. Experimental tests based on a scaled model were conducted. The main research findings and conclusions are as follows:

(1) Theoretical analysis: a beam-spring model was selected as the theoretical basis to establish the model for the opening segment rings. Considering actual engineering scenarios, the opening behavior between rings was categorized into three deformation modes, and the three possible stress states were analyzed. It was found that in the pure bending state, the position of the neutral axis on the contact surface remains constant. Analytical expressions were derived for the opening amount and maximum annular seam contact pressure under different axial forces and bending moments.
(2) Numerical simulation: using ABAQUS 2022 software, the stress state of the segments during tunnel bending deformation was modeled and analyzed. A nonlinear relationship between the seam opening amount and longitudinal bending moment was obtained under constant axial force. Pressure at equidistant points on the seam contact surface was extracted, showing a decrease at all points except at the maximum contact pressure when the bending moment increased to a certain level due to the proximity of the neutral axis; only the maximum annular seam contact pressure maintained a monotonically increasing nonlinear relationship with the opening amount. The aforementioned results were compared with theoretical analyses, showing highly similar curve trends, thereby validating the correctness of the analytical derivation process and results and proving the feasibility of using multi-point pressure monitoring methods to identify segment opening.
(3) Joint opening identification: a scaled model of the tunnel segments was designed and fabricated, and bending opening experiments were conducted by controlling the loading. Based on data collected from sensors, it was judged that the changes in seam contact pressure during opening were consistent with the finite element simulations, and the relationship curve between the maximum annular seam contact pressure and the opening amount also exhibited nonlinear characteristics. This further verified the correctness of the theoretical sections, including formula derivation and numerical simulations, from a practical perspective. Additionally, an opening amount identification method based on multi-point pressure data was proposed. The method has higher accuracy and sensitivity than using maximum annular seam contact pressure.

## Figures and Tables

**Table 1:** Model parameters for numerical simulation.

Structural Part	Material	Element Type	Dimension	Property
Segment ring	C50 Concrete	C3D8R	Outer ϕ6 m Inner ϕ5.3 m Length 1.5m	Modulus 34.5 GPa Poisson’s ratio 0.2
Joint bolt	Grade 8.8 Steel	B31	M30	Modulus 205 GPa Poisson’s ratio 0.3

**Table 2:** Sensors used in the experiment.

Monitoring Parameter	Sensor	Sensor Picture
Seam contact pressure	Thin film pressure sensor	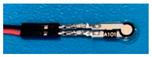
Joint opening	LVDT	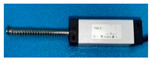
Axial force	Load cell	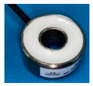

## Data Availability

The raw data supporting the conclusions of this article will be made available by the authors on request.
